# Genetic attributes of midwife toad (*Alytes obstetricans*) populations do not correlate with degree of species decline

**DOI:** 10.1002/ece3.677

**Published:** 2013-07-22

**Authors:** Ursina Tobler, Trenton W J Garner, Benedikt R Schmidt

**Affiliations:** 1 Institute of Evolutionary Biology and Environmental Studies, University of ZurichWinterthurerstrasse 190, CH-8057, Zurich, Switzerland; 2 KARCHPassage Maximilien-de-Meuron 6, CH-2000, Neuchâtel, Switzerland; 3 Institute of Zoology, Zoological Society of LondonRegent's Park, NW14RY, London, U.K

**Keywords:** *Alytes obstetricans*, genetic diversity, geographic variation, population decline, population structure

## Abstract

Genetic diversity is crucial for long-term population persistence. Population loss and subsequent reduction in migration rate among the most important processes that are expected to lead to a reduction in genetic diversity and an increase in genetic differentiation. While the theory behind this is well-developed, empirical evidence from wild populations is inconsistent. Using microsatellite markers, we compared the genetic structure of populations of an amphibian species, the midwife toad (*Alytes obstetricans*), in four Swiss regions where the species has suffered variable levels of subpopulation extirpation. We also quantified the effects of several geographic factors on genetic structure and used a model selection approach to ascertain which of the variables were important for explaining genetic variation. Although subpopulation pairwise *F*_ST_-values were highly significant even over small geographic scales, neither any of the geographic variables nor loss of subpopulations were important factors for predicting spatial genetic structure. The absence of a signature of subpopulation loss on genetic differentiation may suggest that midwife toad subpopulations function as relatively independent units.

## Introduction

The maintenance of population genetic diversity is of crucial importance for long-term population persistence (Frankham 2005; Evans and Sheldon 2008) Reducing the number of migrants into a recipient population is a proven mechanism for reducing its effective population size and causing a reduction in measurable genetic diversity through genetic drift (Frankham 1995, 2005). Immigration rate is expected to scale with the availability of source populations, so population loss should have a direct and measurable effect on the genetic variability and genetic similarity of the remaining populations. Given sufficient time (i.e. 20–50 generations; Anderson et al. 2010), in areas where population loss is significant, among-population differentiation should be greater and within-population genetic variability lower than in areas where local extirpation has been less frequent (Lande et al. 1998; Frankham 2005). While the theory is well-developed (Gilpin 1991), empirical evidence in support of theory is inconsistent (Keyghobadi 2007). Populations of taxa that were classified as threatened by the International Union for Conservation of Nature (IUCN) and have experienced significant population loss exhibit lower population genetic diversity than do unthreatened sister taxa (Spielman et al. 2004; Evans and Sheldon 2008). However, other studies have found no such relationship (Gibbs 2001; Keyghobadi 2007). Determining why there are inconsistencies is hampered by the sampling design of many studies of population genetics and population loss. Most are unreplicated, conducted at a single site or within a single geographic area (Matocq and Villablanca 2001) and hence may be unrepresentative of the general relationship between rapid decline and loss of genetic diversity. A better approach would be comparative, measuring the consistency of the relationships between population loss and genetic variability in replicated regions.

The global decline of amphibians has resulted in the loss of amphibian populations over a matter of decades, but declines are not homogeneously distributed (Houlahan et al. 2000; Stuart et al. 2004). The amphibian extinction crisis is certainly one of the most topical conservation issues of our generation, but it could also be viewed as an opportunity for investigating the links between decline and population genetic structure. Previous studies have illustrated a relationship between population loss and genetic diversity on a limited geographic scale (Hitchings and Beebee 1997, 1998; Beebee 2005). We know of no study that has made an explicit attempt to relate population loss to its effects on gene flow and population genetic variability in a replicated fashion.

Adopting the classic definitions of Wright (1965) from hereon, a region across which amphibian breeding ponds are distributed is equivalent to a population and each pond within a population to a subpopulation. A region refers to a geographic area harboring a number of subpopulations, but not necessarily representing a closed population without genetic exchange with other such populations. A suitable amphibian species to study the link between decline and genetic structure would have the following traits: (1) discrete subpopulations; (2) data available on subpopulation persistence across comparable populations; and (3) variable rates of subpopulation extinction across comparable populations. Such a model exists in the common midwife toad, *Alytes obstetricans*, in Switzerland, where the distribution of the species is well known (Grossenbacher 1988; Borgula and Zumbach 2003). *A. obstetricans* exhibits a complex life history with an aquatic larval stage and a terrestrial adult stage, so breeding ponds can be treated as defined subpopulations.

*Alytes obstetricans* has suffered strong declines in Switzerland: The species was first red-listed in 1982 (Hotz and Broggi 1982) because population declines were already observed in Switzerland in the 1960s (Escher 1972). The most recent update of the Swiss amphibian red list showed that since the mid-1980s ∼50% of subpopulations have been extirpated (Schmidt and Zumbach 2005). Therefore, *A. obstetricans* is categorized as “endangered” in the most recent Swiss Red List (Schmidt and Zumbach 2005). Very few colonizations of unoccupied ponds have been observed and Swiss *A. obstetricans* subpopulations are small (Borgula and Zumbach 2003; Schmidt and Zumbach 2005; Tobler et al. 2012). Given that *A. obstetricans* has a generation time of 1–2 years (Böll et al. 2012), >20 generations have passed since the onset of declines, sufficient time for subpopulation loss to have affected genetic structure (Anderson et al. 2010). Rate of subpopulation loss has varied among populations and in most cases subpopulation extirpation cannot be attributed to habitat loss (Borgula and Zumbach 2003; Schmidt and Zumbach 2005).

**Figure 1:**
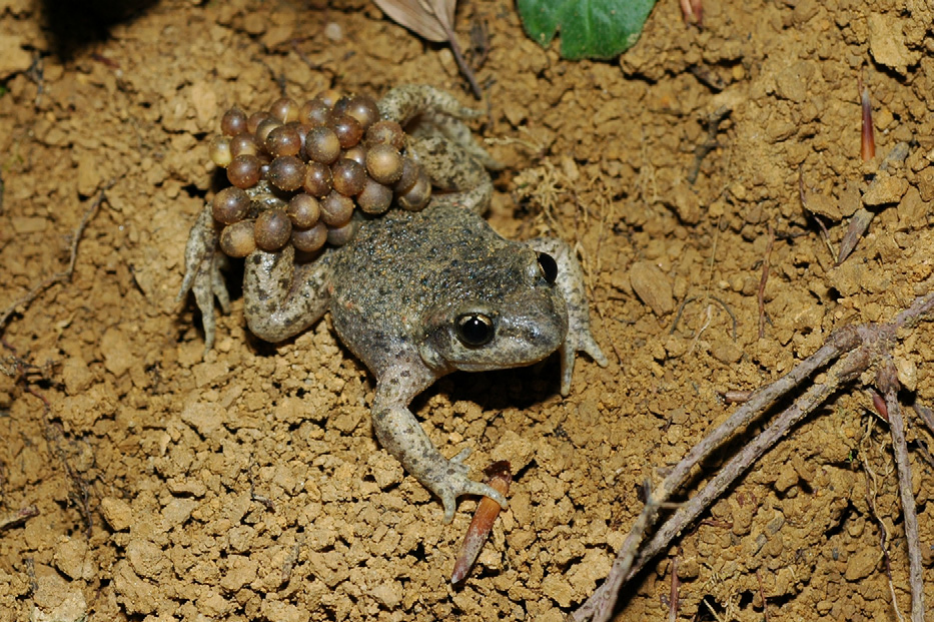
Male common midwife toad, *Alytes obstetricans*, carrying egg strings. Photograph by Ursina Tobler.

In this study we take advantage of existing knowledge regarding the spatial distribution of recent extirpations of *A. obstetricans* (Fig. 1) subpopulations and ask whether population genetic structure and diversity measured in subpopulations and populations varies and if this variation can be attributed to among-population variation in rates of recent subpopulation loss. To do this, we sampled four regions where the common midwife toad is found and where quantitative evidence of variation in subpopulation loss is available. We used microsatellite polymorphisms to measure within-subpopulation and within-population genetic variability, as well as gene flow among subpopulations. We distinguished between the effects of subpopulation loss and region-specific characteristics on genetic diversity and population size by modeling whether other factors such as geographic connectivity, elevation, or location close to a stream contributed to the observed genetic patterns. We expected that the loss of subpopulations that occurred over a time frame of roughly 20 generations would be paralleled by decreasing population sizes and have caused an increase in genetic differentiation among subpopulations and a reduction in genetic diversity due to increased drift (Lande 1988). We predicted similar consequences due to the effect of weaker geographic connectivity (Cushman 2006). Because suitable habitat becomes scarcer towards the distribution limits of a species, higher elevation subpopulations should also be more isolated than lowland ones so we predicted similar effects as those resulting from range periphery on high-elevation subpopulations (Giordano et al. 2007). Thus, we expected reduced genetic diversity and heterozygosity, and increased subpopulation differentiation at higher elevation. Finally, because midwife toads sometimes occur in stream or inhabit ponds near streams (Barandun 2007), we also assessed the effect of streams on the genetic structure. Streams may act as corridors for gene flow either downstream (tadpoles) or upstream (adults; Morrissey and de Kerckhove 2009; Grant et al. 2010; Mullen et al. 2010), hence we expected weaker genetic differentiation among subpopulations along identical catchments (Table 1).

**Table 1:** Predictions of how factors are expected to affect allelic richness, expected heterozygosity, and *F*_ST_, and the observed effect on the genetic measures

Factor	Levels	Prediction	Observed effect
Decline	3 (0, 1,2)	Low gene flow among subpopulations	1. No difference in allelic richness
		1. Decrease in allelic richness due to random loss of alleles through genetic drift	2. No difference in *H*_e_
		2. Only slight reduction in *H*_e_ because rare alleles lost by drift contribute little to *H*_e_	3. No difference in *F*_ST_
		3. Stronger genetic differentiation among subpopulations due to increased drift	
Geographic connectivity	45 (mean pairwise geographic distances)	Low gene flow among subpopulations, but less strong effects than under decline	1. No difference in allelic richness
		1. No or only slight decrease in allelic richness due to random loss of alleles through genetic drift	2. No difference in *H*_e_
		2. No reduction in *H*_e_	3. No difference in *F*_ST_
		3. Increased genetic differentiation	
Elevation	45 (elevation of study sites)	Larger distance between less suitable habitat patches	1. Increase in allelic richness with increasing elevation
		1. Decrease in allelic richness due to random loss of alleles by genetic drift in smaller populations at high elevation	2. No difference in *H*_e_
		2. Slight decrease in *H*_e_ because *H*_e_ degrades more slowly than allelic richness	3. No difference in *F*_ST_
		3. Stronger genetic differentiation among subpopulations due to lower connectivity	
Location along stream	2 (0, 1)	Increased connectivity	1. Increase in allelic richness with increasing elevation
		1. Increased or equal allelic richness due to enhanced gene flow along streams	2. No difference in *H*_e_
		2. No difference in *H*_e_	3. No difference in *F*_ST_
		3. Lower *F*_ST_	

## Methods

### Study populations

**Figure 2:**
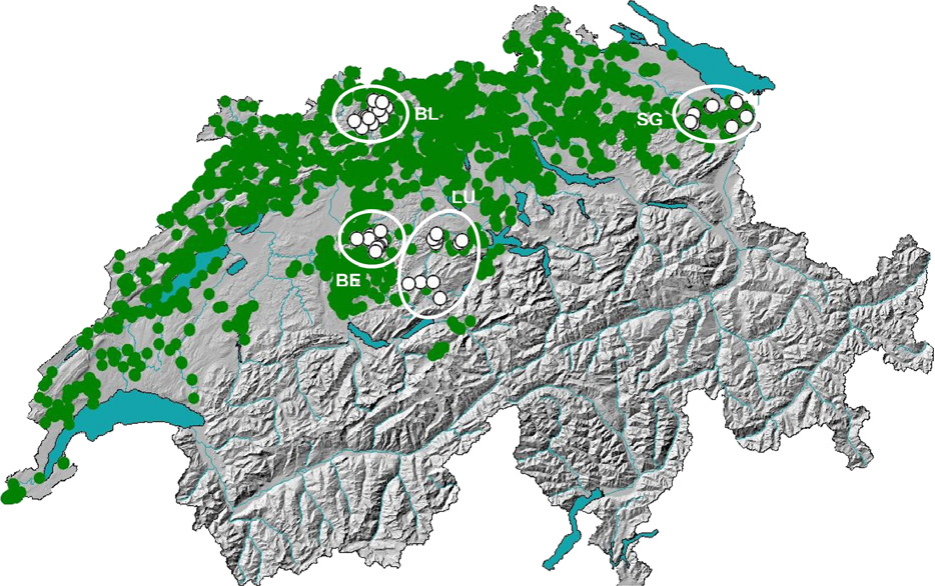
Map of Switzerland showing the distribution of *Alytes obstetricans* (green dots) and the location of the study populations (white dots). Data sources and copyright: Swisstopo and KARCH (Koordinationsstelle für Amphibien- und Reptilienschutz in der Schweiz, http://www.karch.ch).

We collected tissue samples for genetic analyses from 45 subpopulations clustered within four populations in Switzerland during spring and summer 2007: Baselland (BL, 15 subpopulations), Bern (BE, 11 subpopulations), Lucerne (LU, 11 subpopulations), and St. Gallen (SG, 8 subpopulations; Fig. 2). We chose these populations based on available knowledge on midwife toad subpopulation trends (Table 2) and the fact management practices have aimed to increase and improve the quantity and quality of breeding sites in all four populations. Therefore, there was no net loss of available breeding sites for *A. obstetricans* across our study system.

**Table 2:** Characteristics of the four study regions

	Region			
	
	BE	BL	LU	SG
Number of populations	11	15	11	8
Declines	Moderate	None	Strong	Strong
Time frame	1970–2003	1980–2010	1980–2003	1980–2003
% gains	+8%	+41%	+6%	+0%
% losses	−29%	−8%	−55%	−77%
Elevation of study sites (mean [range])	790 m.a.s.l. (590–940)	485 m.a.s.l. (400–590)	878 m.a.s.l. (590–1540)	543 m.a.s.l. (450–680)
Number of populations in/along streams	0	0	6	2
Distance among subpopulations (mean [range])	4.3 km (0.6–9.0)	5.7 km (0.9–13.2)	13.1 km (1.2–25.9)	11.1 km (0.9–20.8)

BL is located in the Jura Mountains and supports a comparatively dense network of *A. obstetricans* subpopulations. The mean Euclidian distance between all known breeding sites is 0.89 km (4.3 km among study subpopulations) and 74 subpopulations of *A. obstetricans* were detected from 1981 to 1993 (Schmidt et al. 2010). Subpopulations of *A. obstetricans* in BL are relatively persistent: 51 of 74 known subpopulations were revisited in 2009 and species presence was confirmed at 45 of these. In addition, many hitherto unreported subpopulations were detected (Schmidt et al. 2010). Most subpopulations are found in man-made ponds. In BE, subpopulations of *A. obstetricans* are also relatively densely distributed (mean distance among subpopulations: 1.3 km; B. Lüscher, pers. comm.; 4.3 among study subpopulations) and declines have been moderate (of 149 total subpopulations known since 1970, 43 went extinct until 2003; Ryser et al. 2003). Colonization of new ponds is reported, but at a low rate (only 12 new subpopulations were detected as of 2003; Ryser et al. 2003). In BE, *A. obstetricans* is found predominantly in man-made ponds.

Lucerne is a pre-alpine region where the distances between subpopulations are relatively large (mean Euclidian distance of 17.7 km among subpopulations; A. Borgula, pers. comm.; 13.1 km among study subpopulations). Relatively more subpopulations of *A. obstetricans* in LU have been lost (51 subpopulations reported in 1980 down to 23 reported in 2002) than in BL and BE. There are only three reports of colonization of new subpopulations (Borgula and Zumbach 2003). Breeding habitats for *A. obstetricans* in LU are man-made ponds located in pre-alpine and alpine meadows, and one stream relatively unchanged by human activity. BE and LU are adjacent but are separated from each other by prealpine topography and are part of different drainage basins. The colonization of prealpine areas after the last glaciation followed the retreat of the ice starting from the lowlands (Hewitt 1996; Martínez-Solano et al. 2004), hence we consider LU and BE genetically distinct populations, and test this (see below). SG is the second region where *A. obstetricans* has experienced severe subpopulation loss and is also a prealpine region. Since 1980, *A. obstetricans* is absent from 68 of 118 recorded subpopulations and another 23 are potentially extirpated. No colonizations are reported (Barandun 2004). Breeding habitats in SG are streams and man-made ponds; mean distance among subpopulations in this region is 19.8 km (J. Barandun, pers. comm.; 11.1 km among study subpopulations). Both LU and SG are located on the periphery of the species distribution in Switzerland (Fig. 2).

**Figure 3:**
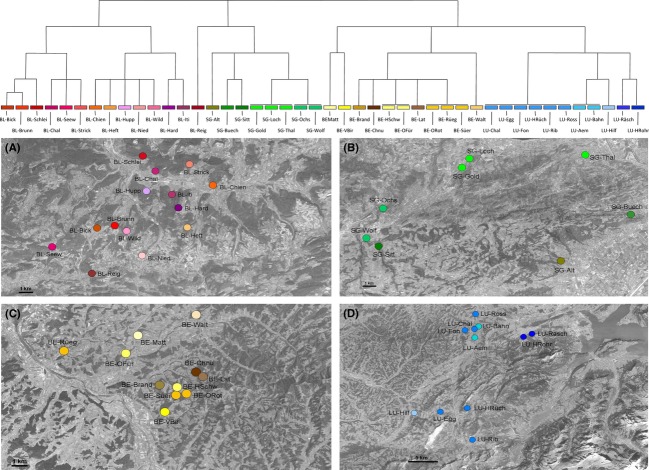
Results of the genetic clustering analysis in STRUCTURE. The cladogram on top shows how populations were split into clusters along the step-wise analyzing process. Every cluster identified on a hierarchical level was subjected to a new STRUCTURE analysis until STRUCTURE was unable to split clusters further. Populations with equal colors form one single cluster. Maps A to D show the geographic location and the cluster assignment of the populations within regions. (A) BL, (B) SG, (C) BE, (D) LU.

In each population we sampled 8–15 ponds previously reported to harbor *A. obstetricans* subpopulations. In LU, we sampled two clusters of subpopulations of which the southernmost was located at the distribution border. Subsequent analyses confirmed that, although no known populations exist between the southern and northern cluster, these clusters could still be treated as one population (see below; Fig. 3). The measure of population differentiation we used is relatively insensitive to unsampled populations (Koen et al. 2013) and the populations that were not sampled were small and most likely do not contribute many migrants. Hence, we do not expect that the presence of unsampled populations caused bias in our estimates of population differentiation and isolation-by-distance. We caught tadpoles by dip-netting and collected tissue for DNA extraction by cutting off less than 3 mm from the tail tip. We sought to sample a minimum of 25 tadpoles per site. All tadpoles were released into their source ponds immediately thereafter. Standard hygiene protocols to avoid the spread of infectious diseases were followed during field work (Schmidt et al. 2009).

### Microsatellite development

Microsatellite primers were commercially developed by ecogenics GmbH (Zurich, Switzerland) using *A. obstetricans* tissue samples from central Spain, France and Switzerland. An enriched library was made from the DNA of one Swiss *A. obstetricans*: size selected genomic DNA was ligated to SAULA/SAULB-linker (Armour et al. 1994) and enriched by magnetic bead selection with biotin-labeled (GT)_13_, (CT)_13_, (GATA)_7_, (GTAT)_7_, (ACAG)_7_, and (GCGT)_7_ oligonucleotide repeats (Gautschi et al. 2000). Of 1893 recombinant colonies screened, 241 gave a positive signal after hybridization. Plasmids from 187 positive clones were sequenced and primers were designed for 29 microsatellite inserts, of which 21 were tested for polymorphism. We selected a set of 12 primers that exhibited clear and reliable amplification, polymorphism and no evidence of null alleles in preliminary tests for generating population genetics data (Table 3).

**Table 3:** Microsatellite primer sequences, repeat types, size range, allele numbers, and concentrations used in multiplex PCR reactions

Multiplex	Locus		Primer sequence 5′-3′	Modification 5′	Repeat type^1^	Size bp EU^2^	No. of alleles EU^2^	Size bp CH^3^	No. of alleles CH^3^	Concentration per reaction
1	Alyobs3	F	CCAACATGTTCACTTTATAGAGCAG	Yakima Yellow	(TATC)_28_	203–272	11	182–279	18	1.25 μM
		R	GGAACCTTGAATCTCGAAAGC							1.25 μM
1	Alyobs4	F	TTTTCCCTTGCTAAATCCTCAG	ATTO565	(CTGT)_11_	123–167	7	134–143	3	0.75 μM
		R	AAAGTGTTGATGCACATTTTCC							0.75 μM
1	Alyobs7	F	AAGGACGTGCTTCTATCTGC	FAM	(TATC)_16_(TG)_3_(TA)_3_(TC)(TA)_4_	116–268	13	124–210	14	1.25 μM
		R	AGTTCGCACACATTACATTGC							1.25 μM
1	Alyobs28	F	CCAGTGCTGTGGTTTTCTCA	Yakima Yellow	(GT)_13_(GA)_3_(GTGA)_3_	100–136	10	103–109	3	1.88 μM
		R	AAATATCAAGAGCCTTAGCTAACATTT							1.88 μM
2	Alyobs8	F	TGAGGGGTCAGTGGAAGATATAC	FAM	(ACAG)_11_(AG)_2_(ACAG)(ATAG)_23_	223–332	13	158–340	24	2.00 μM
		R	GGACAAATTCCAGCATAAAGAAC							2.00 μM
2	Alyobs16	F	CAATGGCTGTACACAAGGAAAC	FAM	(GT)_17_	134–150	9	132–146	7	7.80 μM
		R	CCTATAGAAATGTAAACATGCACAC							7.80 μM
3	Alyobs17	F	TTCTCTTCAGCTGGGCAATC	Yakima Yellow	(GT)_13_	137–161	6	148–165	6	2.50 μM
		R	TGGAACTGAAGAGCGAGGAC							2.50 μM
3	Alyobs19	F	TGAATGTGCCGGTGAAGAC	FAM	(GT)_12_	69–103	8	76–82	3	2.50 μM
		R	AAACACATATGAACAGGTGAAAAGAG							2.50 μM
3	Alyobs20	F	GATGCAGCACATTTCTGAGC	ATTO565	(GT)_12_	96–111	4	113–113	1	0.42 μM
		R	GGTGCATCTGCCATAGTGTG							0.42 μM
3	Alyobs23	F	TGCAGAGCTCAGCCACTTAG	ATTO550	(GT)_13_	206–244	5	207–215	4	0.50 μM
		R	TGACCAATCCAATCATCCAG							0.50 μM
3	Alyobs24	F	TCCTCAAAATCTTGTGATGTGC	ATTO550	(CA)_28_	79–134	13	102–147	17	0.50 μM
		R	ATGGCCAGATGTCCCAATAC							0.50 μM
3	Alyobs25	F	CCTTCTGTCTACCTTGTCATATTTCC	ATTO565	(GT)_16_	138–160	6	154–167	5	0.50 μM
		R	AAAGCGACTAATACAGAACACTGC							0.50 μM

1 Based on sequenced clone.

2 Based on 15 individuals from France, Spain, and Switzerland.

3 Based on 1389 individuals from Switzerland.

### Microsatellite amplification

We extracted DNA from tail clips using the BioSprint 96 DNA Blood Kit (Qiagen, Hombrechtikon, Switzerland), following the protocol for tissue extraction. Polymerase chain reactions (PCR) were performed with fluorescent-labeled primers in three separate multiplexes (Table 3). Each well contained 1.25 μL Multiplex PCR Kit (Qiagen), the respective primer volumes (Table 3) and 1 μL of template DNA. Forward primers were color-labeled to allow multiplexing. PCRs were carried out on a TC-412 Thermal Cycler (Barloworld Scientific, Staffordshire, UK) with polymerase activation at 95°C for 15 min, followed by 33 cycles (multiplex 1) or 30 cycles (multiplexes 2 and 3) of denaturing for 0.30 min at 94°C, annealing for 1.30 min at 52°C (multiplex 1) or 56°C (multiplexes 2 and 3) and extension for 1.00 min at 72°C, followed by a final extension for 30 min at 60°C. PCR products were run on an ABI 3730 Avant capillary sequencer (Applied Biosystems, Rotkreuz, Switzerland) with internal size standard GeneScan-500 LIZ; peaks were visually scored using GENEMAPPER 3.7 (Applied Biosystems 2004).

### Statistical analysis

Microsatellite loci were tested for the presence of null alleles, stuttering and allelic dropout of larger alleles using MICROCHECKER (Van Oosterhout et al. 2004). Because of the potential presence of siblings in our dataset (Goldberg and Waits 2010), we tried to identify siblings using COLONY 3.1 (Wang 2004). We did not find within-population sibship structure, indicating there are no confounding effects of relatedness on our data (J. Wang, pers. comm.). We used ARLEQUIN 3.1 (Excoffier et al. 2005) to test for linkage disequilibrium and deviations from Hardy–Weinberg (HW) equilibrium and tested all microsatellites for evidence of locus-specific selection using the program FDIST (Beaumont and Nichols 1996).

Measures of genetic diversity per subpopulation and population (observed heterozygosity [*H*_o_], expected heterozygosity [*H*_e_], and the frequency of private alleles) were calculated using GENETIX 4.05.2 (Belkhir et al. 1996) and a sample-size corrected estimate of allelic richness (A) was obtained using FSTAT 2.9.3.2. To obtain an estimate of number of breeding individuals per population (*N*_b_), we ran the programs ONeSAMP (Tallmon et al. 2007) with prior information between 2 and 100 for each population, and Colony (Wang 2004) assuming a polygamous breeding system for both males and females, and using the full likelihood model with medium precision and no prior information. As both analyses returned very similar values, we only report the results obtained using Colony.

To test whether the regions could be treated as distinct genetic units, we performed a Bayesian clustering analysis using the software STRUCTURE (Pritchard et al. 2000). To infer the number of clusters in our data, we used the Δ*K* method proposed by Evanno et al. (2005), modified using a stepwise approach according to Coulon et al. (2008). Specifically, we repeated the estimation of the number of clusters with the Δ*K* method on each of the *K* groups inferred in the previous step. We repeated this process until the number of clusters inferred was 1, or individuals from any single pond were split. Each simulation was run with a burn-in period of 50,000 iterations and a sampling period of 250,000 iterations. For every simulation step, we set the bounds of *K* from 1 to 15 and repeated the simulation for each *K* 10 times.

Because a proportion of young-of-the-year *Alytes* tadpoles hibernate as tadpoles and only metamorphose in the year after hatching (Thiesmeier 1992), we first calculated *F*_ST_ values treating overwintered tadpoles from 2006 and young-of-the-year tadpoles from 2007 as separate units (“populations” in the terminology of the program) in all ponds where data on two cohorts was available. This analysis tested whether reproduction among cohorts was strongly skewed towards a few parental individuals (Savage et al. 2010). Because all *F*_ST_-values between cohorts were not significant (results not shown), we assumed that our measures of genetic diversity with all samples from a subpopulation pooled were representative. We next calculated global *F*_ST_-values per region and pairwise *F*_ST_-values of subpopulations within regions as a measure of genetic population structure among and within regions and tested the significance of pairwise *F*_ST_ within regions with an exact test with 1000 permutations (Excoffier et al. 2005). We tested for isolation by distance (IBD) across and within populations using *F*_ST_/(1−*F*_ST_)-transformed *F*_ST_ values and log-transformed Euclidian distances (Excoffier et al. 2005). To determine at what degree genetic variation was distributed within and among subpopulations and populations, we used analysis of molecular variance (AMOVA) implemented in Arlequin 3.1 (Excoffier et al. 2005).

To test our hypotheses regarding the effects of severity of declines, geographic connectivity, elevation, or location near a stream on population size and differences in genetic structure we combined linear mixed effects models with an Akaike's information criterion (AICc) based inference framework (Burnham and Anderson 2002).

Severity of subpopulation loss was classified as “none,” “moderate,” or “strong” (Table 2). We extracted data on elevation and pairwise Euclidian distances among subpopulations within a region from the national map (1:25,000; Swisstopo 2003–2009). Geographic isolation within a population was defined as mean pairwise Euclidian distance of a subpopulation to all other subpopulations in the same region. We considered subpopulations as stream subpopulations if they were within 200 m of a stream or when the breeding site was a stream. We used A, *H*_e_, and the mean subpopulation *F*_ST_ as response variables in separate linear mixed effects models; we did not use all variables of genetic diversity that are commonly used in population genetic studies because all measures of allelic diversity (number of private alleles, fixed loci, and A) were correlated (all *r* > 0.45), and the same was true for *H*_e_ and *H*_o_ (*r* = 0.89). We calculated a mean subpopulation *F*_ST_ as the mean of all pairwise *F*_ST_ values of a subpopulation to all other subpopulations within the same population.

Our independent variables, all defined above, were (1) severity of subpopulation loss, (2) elevation, (3) location close to a stream, and (4) geographic isolation within a population. We used an intercept-only model as the null model. We included one main effect per model only. Population was defined as a random effect to accommodate non-independence of the sub-populations in all models (Rhodes et al. 2009) We standardized elevation and isolation for the use in linear mixed effects models using a z-transformation. After fitting the models, we used spline correlograms to investigate auto-correlation in the data (Rhodes et al. 2009).

We ranked models based on AICc for small sample sizes (Burnham and Anderson 2002) and report parameter estimates for models within 2 ΔAICc units of the best model. Model assessment was also based on inspection of the parameter estimates and their confidence intervals; that is, only parameters of which the confidence interval did not include 0 were considered important (Burnham et al. 2011). All linear mixed models were fitted to the data using maximum likelihood in R 2.8.1. using the package lme4 (R Development Core Team 2008; Bates et al. 2011).

## Results

### Genetic diversity

We did not detect null alleles, large allele dropout or stuttering at any microsatellite locus (all *P* > 0.05). Locus Alyobs20 was monomorphic in all studied subpopulations and excluded from further analyses. We found no deviations from HW equilibrium after Bonferroni correction and very little and inconsistent linkage at few markers in few subpopulations. With the application of a conservative significance level of *P* = 0.001, 24 signatures of linkage were found in a total of 4730 comparisons (11 markers times 43 subpopulations); linked markers were found in 8 of the 43 subpopulations. This is further indication that the (possible) presence of siblings in the genotype data did not negatively affect our results (Rasmussen 1979). Four markers were identified as being potentially non-neutral. Alyobs23 was designated as a candidate for directional selection, while Alyobs3, Alyobs4, and Alyobs8 were identified as candidates for balancing selection (Beaumont and Nichols 1996). Excluding these markers from the calculation of our measures of genetic diversity estimates did not alter the model selection results (results not shown) hence we concluded that potential non-neutrality did not bias our results. We thus report the estimates of genetic diversity based on all 11 polymorphic markers. Average allelic richness (A) within subpopulations was low. Six loci, on average, were fixed in every subpopulation and we detected on average one private allele in every second subpopulation (22 private alleles in 45 populations; Table 4). Average H_e_ was also low and the difference between H_e_ and H_o_ never exceeded 0.12 (Table 4). Mean breeding population size was low with 16.1 individuals, ranging from 8 to 31 breeders (Table 4).

**Table 4:** Measures of genetic diversity for all subpopulations

Population	Subpopulations	Individuals sampled	Total no. of alleles	No. of fixed loci	No. of private alleles	*A*	*H*_e_	*H*_o_	Mean *F*_ST_^1^	*N*_b_ (95% CI)
BE	BE-Brand	62	25	8	0	2.17	0.21	0.22	0.248	17 (10–34)
	BE-Chnu	47	25	7	0	2.17	0.27	0.31	0.253	15 (8–30)
	BE-HSchw	15	23	8	0	2.00	0.25	0.36	0.199	8 (4–22)
	BE-Lat	23	18	4	0	1.58	0.20	0.17	0.253	13 (7–31)
	BE-Matt	52	26	5	1	2.25	0.21	0.22	0.342	19 (10–38)
	BE-OFür	30	21	5	0	1.83	0.25	0.28	0.173	15 (8–31)
	BE-ORot	35	28	7	0	2.42	0.29	0.30	0.147	16 (9–34)
	BE-Rüeg	6	21	5	1	1.83	0.24	0.36	0.189	15 (5–inf.)
	BE-Süer	20	21	5	0	1.83	0.26	0.33	0.176	8 (4–19)
	BE-VBir	50	31	9	0	2.67	0.29	0.30	0.133	23 (14–42)
	BE-Walt	61	28	5	1	2.42	0.22	0.24	0.247	18 (10–35)
	Mean BE		25.6	6.4	0.4	2.22	0.25	0.28	0.215	15.2
BL	BL-Bick	51	38	7	2	3.25	0.33	0.32	0.205	31 (19–52)
	BL-Brunn	19	27	6	0	2.33	0.33	0.35	0.228	15 (7–33)
	BL-Chal	31	37	6	0	3.17	0.27	0.27	0.242	20 (11–39)
	BL-Chien	24	32	6	2	2.75	0.28	0.28	0.237	12 (6–30)
	BL-Hard	21	30	6	0	2.58	0.28	0.31	0.234	14 (8–30)
	BL-Heft	57	37	6	1	3.17	0.31	0.33	0.190	30 (18–50)
	BL-Hupp	20	42	7	0	3.58	0.40	0.41	0.177	23 (12–46)
	BL-Iti	52	28	7	2	2.42	0.20	0.20	0.324	21 (12–39)
	BL-Nied	27	39	8	2	3.33	0.34	0.33	0.181	18 (10–37)
	BL-Reig	48	24	6	0	2.08	0.26	0.26	0.398	20 (11–38)
	BL-Schlei	45	36	7	0	3.08	0.36	0.36	0.204	23 (13–42)
	BL-Seew	25	24	4	2	2.08	0.24	0.26	0.276	14 (7–30)
	BL-Stra	11	29	7	0	2.50	0.32	0.35	0.268	12 (6–34)
	BL-Strn	47	39	9	2	3.33	0.32	0.32	0.246	16 (9–31)
	BL-Wild	14	18	3	0	1.58	0.15	0.16	0.431	8 (4–22)
	Mean BL		33.3	6.8	1.0	2.86	0.29	0.30	0.256	18.5
LU	LU-Aem	16	23	7	0	2.00	0.33	0.33	0.222	17 (9–41)
	LU-Bahn	23	25	7	0	2.17	0.34	0.32	0.184	21 (11–43)
	LU-Chal	24	21	6	0	1.83	0.18	0.19	0.215	13 (7–30)
	LU-Egg	21	30	7	1	2.58	0.29	0.29	0.157	16 (8–34)
	LU-Fon	20	27	6	0	2.33	0.28	0.31	0.194	12 (6–30)
	LU-Hilf	24	28	7	0	2.42	0.31	0.35	0.155	19 (10–38)
	LU-HRohr	25	27	6	0	2.33	0.22	0.22	0.271	17 (9–38)
	LU-HRüch	24	29	6	0	2.50	0.25	0.26	0.201	19 (10–36)
	LU-Räsch	22	22	5	0	1.92	0.25	0.30	0.325	10 (5–36)
	LU-Ribi	32	33	6	1	2.83	0.31	0.32	0.155	18 (10–38)
	LU-Ross	24	25	6	0	2.17	0.23	0.24	0.186	12 (6–28)
	Mean LU		26.8	6.4	0.2	2.31	0.28	0.29	0.206	15
SG	SG-Alt	26	27	7	1	2.33	0.33	0.36	0.218	16 (8–34)
	SG-Buech	25	22	5	0	1.92	0.19	0.19	0.316	13 (7–30)
	SG-Gold	21	26	7	0	2.25	0.36	0.38	0.202	10 (5–28)
	SG-Loch	17	27	7	0	2.33	0.30	0.32	0.203	14 (7–31)
	SG-Ochs	21	33	7	2	2.83	0.34	0.35	0.163	14 (7–31)
	SG-Sitt	28	20	5	0	1.75	0.18	0.21	0.299	12 (6–26)
	SG-Thal	7	19	6	0	1.67	0.25	0.27	0.264	11 (4–35)
	SG-Wolf	20	32	6	1	2.75	0.28	0.30	0.187	17 (9–38)
	Mean SG		25.6	6.3	0.5	2.23	0.28	0.30	0.231	13.4

1 mean pairwise *F*_ST_ with respect to all other populations within the same population.

A, allelic richness (sample size corrected); *H*_e_, expected heterozygosity; *H*_o_, observed heterozygosity; *N*_b_, number of breeding individuals.

With only one exception we could assign subpopulations to the correct population in the first step of the STRUCTURE analyses (Figure 3). This initial analysis only split the samples into three clusters, with two of the populations (BE and LU) grouped together. As well, subpopulation BL-Reig, located geographically in BL, clustered with SG. This wrong assignment is probably due to a lack of power with only ∼2 alleles per locus: Out of 24 different alleles at 11 markers in BL-Reig, seven are shared between BL-Reig and the other BL subpopulations, and only one is shared between BL-Reig and the other SG subpopulations, but not with the rest of BL. The other 16 are shared among BL, BL-Reig, and SG. Given the higher accordance of BL-Reig alleles with other BL subpopulations than with SG, it is unlikely that the assignment of BL-Reig to the SG region is due to the release of individuals from SG at the site. Subsequent analysis of the first-level clusters split BE and LU into two separate groups and split BL-Reig from the SG cluster. Further reanalyses generated a total of 32 clusters (Fig. 3).

**Figure 4:**
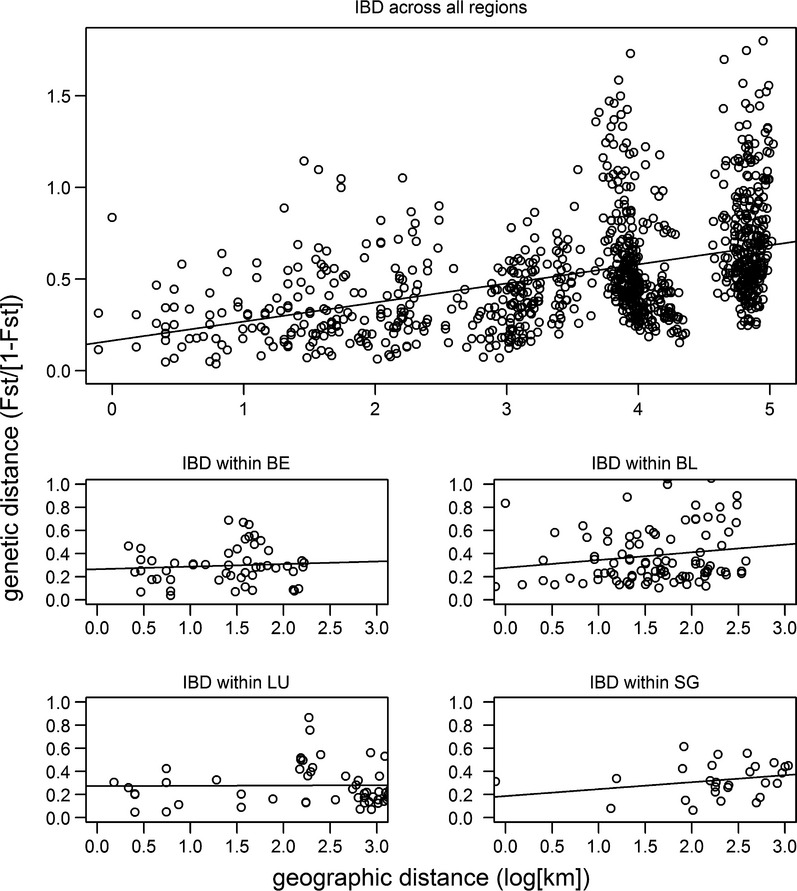
Pairwise *F*_ST_ values plotted against pairwise distances between subpopulations (isolation by distance [IBD]). Top graph: IBD across all regions; four lower graphs: IBD within regions.

Global *F*_ST_ estimated for each of the four populations were within a similar range (BE: 0.239, BL: 0.261, LU: 0.209, SG 0.232) and the overall, global *F*_ST_ was 0.352. Pairwise *F*_ST_ values among subpopulations within populations ranged from 0.035 to 0.534 (BE: 0.035–0.408; BL: 0.093–0.534; LU: 0.045–0.464; SG: 0.059–0.381) and all were significantly different from zero. IBD was highly significant when the entire data set was included in a single analysis (*r* = 0.435, *P* < 0.001) but within populations IBD was not significant for BE (*r* = 0.121, *P* = 0.285), BL (*r* = 0.218, *P* = 0.063), and LU (*r* = 0.010, *P* < 0.482), and only marginally so for SG (*r* = 0.330, *P* = 0.053; Fig. 4).

The AMOVA showed that genetic diversity was predominantly distributed within subpopulations (62%) but still considerable amounts of variation around 20% each occurred among subpopulations within populations and among populations (Table 5).

**Table 5:** Analysis of molecular variance (AMOVA) of 11 microsatellite loci among the study populations (BE, BL, LU, and SG), among subpopulations within populations, and within subpopulations

Source of variation	df	Sums of squares	% of variation
Among populations	4	1121.8	18.5
Among subpopulations within populations	43	1320.6	19.4
Within subpopulations	1326	4407.6	62.0

We could not derive a single model as the most parsimonious explanation for all measures of genetic variability and structure. Allelic richness was best explained by variation in elevation (Table 6), but the confidence interval of the estimate overlapped with zero, suggesting that elevation did not provide a robust explanation for the observed pattern. The intercept-only model was the AICc-best model for H_e_, suggesting that none of the explanatory variables explained variation in H_e_ well (Table 6). Two other models were within ∼2 ΔAICc values (Table 6), but the confidence interval of the estimates of the coefficients of the explanatory variables in these models included zero (Table 7) indicating that these alternative models are not reliable explanations for the data. *F*_ST_ among subpopulations within populations was best explained by decline (Table 6), but the confidence intervals for the coefficients of the decline effect included zero (Table 7), and, as above, we did not accept this top-ranking model as a good fit. The same was true for two other models that ranked within 2 ΔAICc values of the best model. Inspection of the spline correlograms showed that there was no spatial autocorrelation.

**Table 6:** Model selection results. Models included a single independent variable as a fixed effect and population as a random effect

Model	df	ΔAICc		

		AR	*H*_e_	*F*_ST_
Intercept	3	4.28	0.00	0.47
Decline	6	3.59	1.81	0.00
Elevation	4	0.00	8.00	4.63
Stream	4	5.96	1.87	0.93
Isolation	4	6.55	2.30	2.43

ΔAICc values of models for allelic richness (AR), expected heterozygosity (*H*_e_), and mean population *F*_ST_.

**Table 7:** Parameter estimates (mean [95% CI]) for models within 2 ΔAICc units of the best model for the effects of population, decline, elevation, location near a stream, and geographic isolation

	Model	ΔAICc	Intercept	Decline	Elevation	Stream
AR	Elevation	0.00	2.347 (2.037–2.656)		0.128 (−0.031 to 0.288)	
*H*_e_	Intercept	0.00	0.277 (0.258–0.296)			
	Decline	1.81	0.286 (0.259–0.313)	−0.008 (−0.028 to −0.011)		
	Stream	1.87	0.274 (0.252–0.295)			0.015 (−0.030 ro 0.061)
*F*_ST_	Intercept	0.47	0.231 (0.211–0.251)		−0.004 (−0.009 to 0.000)	
	Decline	0.00	0.252 (0.221–0.284)	−0.019 (−0.042 to 0.003)		
	Stream	0.93	0.237 (0.216–0.259)			−0.035 (−0.085 to 0.015)

Only explanatory variables that were among the best ranking models for at least one genetic measure are included in the table.

## Discussion

We predicted that we would find differences in subpopulation and population genetic structure and diversity that correlated with population declines and connectivity. Specifically, we expected to see differences among subpopulations attributable to different population decline histories and habitat variables expected to influence amphibian dispersal. Although these factors varied among the four regions, we detected no evidence that variation in genetic structure and diversity was influenced by any geographical or historical factor (see also Dudaniec et al. 2012). However, estimates of numbers of breeding adults (*N*_b_) were small and levels of genetic variation were very low. Moreover, private alleles were common in subpopulations, supporting our estimates of small breeding population sizes (*N*_b_). Additionally, we identified strong genetic differentiation among subpopulations even across small geographical scales. Distances of less than 500 m were associated with significant pairwise *F*_ST_-values.

Relatively large pairwise *F*_ST_ values indicate moderate rates of gene flow. A pairwise *F*_ST_ value of 0.2 implies that subpopulations exchange about one migrant per generation. Nevertheless, given the very small breeding population sizes that we estimated (Table 4), drift may have a stronger effect than gene flow and may quickly lead to the loss of rare and immigrant alleles and insignificant IBD (Fig. 4). A high proportion of genetic variation occurring among subpopulations within regions (Table 5) suggests that gene flow is not strong enough to homogenize populations.

The fact that local extirpation did not lead to changes in genetic structure of declining populations can be attributed to different causes. We can rule out insufficient time since declines as an explanation because 20 or more generations have passed since the earliest recorded declines (Böll et al. 2012). One possibility is that extirpations affected predominantly sink subpopulations (Gill 1978; Semlitsch 2000; Hels 2002), and loss of sink subpopulations should not result in a reduction in allelic variation (Hanski and Ovaskainen 2000).

### Conservation implications

Matocq and Villablanca (2001) and Short Bull et al. (2011) pointed out the importance of suitable reference groups when interpreting population genetic data. Our study emphasizes the need for a comparative approach when ascertaining the effect of any threatening process on population genetic parameters. Had we only analyzed the subpopulation genetic structure in populations where strong declines of *A. obstetricans* had been reported, we might have concluded that declines were associated with low levels of genetic diversity and strong subpopulation differentiation. On the basis of this, we might have recommended translocations from other populations to enhance genetic diversity. The inclusion of reference populations where the species has not declined or experienced reduced rates of decline allowed us to show that decline and genetic structure were not associated. Certainly, our results showed that researchers should not simply assume that there will be a genetic signature of species decline and (sub)population loss, even if declines have been ongoing for a sufficient amount of time for genetic signatures of decline to manifest.

There is still cause for concern for common midwife toads in Switzerland based on our, and other, results. Allelic richness and heterozygosity were both extremely low across all four regions and *A. obstetricans* subpopulations in Switzerland are generally composed of relatively few breeding adults (this study and Tobler et al. 2012). Small population size and isolation of subpopulations mean that demographic stochasticity is a potential threat to population survival (Lande 1993). Small and isolated subpopulation sizes coupled with low levels of genetic variability are not ideal situations for long-term subpopulation persistence. Indeed, the fact that in two of our study populations subpopulation loss rate exceeded 50% clearly shows that *A. obstetricans* is a species at risk in Switzerland (Schmidt and Zumbach 2005). Given our analyses of the genetic structure of *A. obstetricans* populations, we believe that translocations to allow for genetic rescue would not be a suitable conservation strategy for this species. Our results showed that most genetic diversity is found within rather than among subpopulations, and that this is the case for stable and declining populations alike. Hence, the translocation of individuals from one subpopulation to another seems unlikely to benefit the long-term survival of subpopulations.

Increasing size (i.e., viability) of subpopulations appears to be the most promising conservation strategy. Larger subpopulations are less prone to environmental and demographic stochasticity and have a higher chance of long-term persistence. Increasing the number of subpopulations, for example, by the creation of new breeding sites, would also benefit long-term survival of populations. However, given the low dispersal rate and the resulting low chance for natural colonizations to occur, conservation management of *Alytes* should not rely solely on this approach. Thus, in the short term, increasing subpopulation size seems the most promising strategy to reduce local extinction risk while increasing connectivity through the establishment of new subpopulations may be a long-term goal.
